# Color and physiochemical attributes of pointed gourd (*Trichosanthes dioica* Roxb.) influenced by modified atmosphere packaging and postharvest treatment during storage

**DOI:** 10.3389/fpls.2022.1016324

**Published:** 2022-10-06

**Authors:** Jahidul Hassan, Farzana Jahan, Md. Mijanur Rahman Rajib, Umakanta Sarker, Ikuo Miyajima, Yukio Ozaki, Sezai Ercisli, Kirill S. Golokhvast, Romina Alina Marc

**Affiliations:** ^1^ Department of Horticulture, Bangabandhu Sheikh Mujibur Rahman Agricultural University, Gazipur, Bangladesh; ^2^ Department of Genetics and Plant Breeding, Bangabandhu Sheikh Mujibur Rahman Agricultural University, Gazipur, Bangladesh; ^3^ Institute of Tropical Agriculture, Kyushu University, Fukuoka, Japan; ^4^ Laboratory of Horticultural Science, Faculty of Agriculture, Kyushu University, Fukuoka, Japan; ^5^ Department of Horticulture, Faculty of Agriculture, Ataturk University, Erzurum, Turkey; ^6^ Siberian Federal Scientific Center of Agrobiotechnology RAS, Centralnaya, Presidium, Krasnoobsk, Russia; ^7^ Food Engineering Department, Faculty of Food Science and Technology, University of Agricultural Sciences and Veterinary Medicine, Cluj-Napoca, Romania

**Keywords:** color, nutrition, packaging, postharvest treatment, pointed gourd, shelf-life

## Abstract

The efficiency of modified atmosphere packaging (MAP) in combination with postharvest treatment on the shelf-life, physiochemical attributes, color, and nutrition of pointed gourd was studied after storing in refrigerated (low temperature, LT) and ambient (room temperature, RT) conditions. Fresh pointed gourd fruits were dipped in NaOCl solution (0.01% w/v) and potassium metabisulphite (KMS) (0.05% w/v), blanched (100°C for 4 min), and then packed in perforated and non-perforated polythene and polypropylene packets of each type and brown paper bags as MAP before storing at LT and RT. Physiochemical attributes, color, and nutrition were measured until the marketable level of acceptance (up to shelf-life) after storage and compared with the untreated and unpacked samples (control). The results showed profound differences among the treatment variables in all the studied dependent parameters regarding the LT and RT storage conditions. Among the treatments, perforated and non-perforated polyethylene (NPE) and polypropylene (NPP) packaging performed well to retain a considerable amount of ascorbic acid, β-carotene, and greenish color (lower L*, high h*) in pointed gourd treated with NaOCl (0.01%) and KMS (0.05%) after storing at LT and RT. Furthermore, the principal component analysis suggested that five major quality attributes (L*, C*, h*, shelf-life, and ascorbic acid) were influenced remarkably in terms of non-perforated polyethylene packaging in combination with KMS treatment both in LT and RT storage conditions. However, perforated polythene and polypropylene in combination with NaOCl responded well in RT but only for the shortest storage life. Thus, a non-perforated polythene package with KMS treatment would be the best solution for retaining market quality acceptance with green color up to the extended shelf-life of 23 and 10 days, respectively, in the refrigerator (LT) and in ambient (RT) storage conditions.

## Introduction

Pointed gourd (*Trichosanthes dioica* Roxb.) is one of the most important fruit-type vegetables belonging to the Cucurbitaceae family. It is thought to be vital or king for higher nutritional and medicinal properties, especially in lowering total cholesterol and blood sugar (Saha et al., 2004; Hassan and Miyajima, 2019a). Although pointed gourd is categorized as non-climacteric, it behaves like climacteric with increased respiration rate after harvest; hence, traditional storage techniques very quickly discard fruits as a consequence of shriveling, skin yellowing, hard seed development, and fungal infection (Koley et al., 2009). In addition, pointed gourd fruit at the green stage has the main edible part with soft seeds after cooking and freshness with adequate moisture; firmness is the most desirable trait for market acceptance (Hassan et al., 2020; Hassan and Miyajima, 2020). Fruit appearance is greatly emphasized because minimum wrinkling or yellowing could lead to losing consumer acceptance even though the fruit is still edible (Hassan and Miyajima, 2019b). The common practice for pointed gourd marketing in Bangladesh and India is bulk packaging in gunny bags followed by moistening with water and storage in unfavorable conditions of high temperature with low humidity, tending to result in a dramatic loss of physiochemical attributes, chlorophyll depletion, and skin hardness with less cell turgidity, thus shortening the postharvest life and market value (Guharoy et al., 2006). Consequently, the seller often resort to trickery by using health-hazardous colors and chemicals like CuSO_4_ to overcome such damage costs (Koley et al., 2009). Therefore, postharvest management of pointed gourd with user-friendly and health-befitting substances still has merits.

Vegetables are an inexpensive and rich source of carotenoids (Sarker et al., 2018a; Sarker et al., 2018b; Sarker et al., 2018c), vitamins, like vitamin A and vitamin C (Sarker and Oba, 2018a; Sarker and Oba, 2018b; Sarker and Oba, 2018c; Sarker and Oba, 2018d; Sarker and Oba, 2018e; Sarker and Oba, 2019a; Sarker and Oba, 2019b; Sarker and Oba, 2019c), mineral elements (Chakrabarty et al., 2018; Sarker et al., 2018; Sarker et al., 2022), protein (Sarker et al., 2014; Sarker et al., 2015a; Sarker et al., 2015b; Sarker et al., 2016; Sarker et al., 2017), dietary fiber (Sarker and Oba, 2020a; Sarker and Oba, 2020b; Sarker et al., 2020; Sarker and Oba, 2020c; Sarker et al., 2020), pigments, phenolics (Sarker and Oba, 2020d; Sarker and Oba, 2020e; Sarker et al., 2022), flavonoids (Sarker and Oba, 2019d; Sarker and Oba, 2020f; Sarker and Oba, 2020g; Sarker et al., 2022), and antioxidants (Sarker and Oba, 2020h; Sarker and Oba, 2021; Hossain et al., 2022; Sarker et al., 2022). However, the physiochemical, color, and nutritional attributes and shelf-life of vegetables for acceptance degrade over prolonged storage duration. Several authors suggested different management strategies including modified atmosphere packaging (MAP) (Maleki et al., 2018), wax coating (Patel et al., 2013), blanching (Sharma and Shrivastava, 2017); use of disinfectants (Feygenberg et al., 2005), hormones (Sen and Sengupta, 2011), and probiotics (Shah and Hashmi, 2020); and improving the storage conditions (Rai et al., 2011) to get rid of physiochemical, color, and nutritional damage as well as to increase the shelf-life of different fruits and vegetables, including pointed gourd (Sahoo et al., 2015). reported that MAP created suitable in-pack conditions with low O_2_ and high CO_2_ along with polypropylene (PP) film that retains the freshness and marketability and prolongs the shelf-life of pointed gourd up to 16 days under refrigerated storage (4–6°C, 45% RH). Even under ambient conditions (23–30°C, 45–75% RH), low-density polyethylene with pinholes lengthens the storage period up to 4 days. Chakraborty et al. (Chakraborty et al., 1991) revealed no shrinkage and lessened yellowing by dipping the pointed gourd into potassium metabisulphite (KMS) solution (1,900 mg/L) for 10 min, whereas (Mohammed and Wickham, 1993) tested sodium hypochlorite (NaOCl) solution for 45 min at 10°C to remove field heat and surface pathogen in pointed gourd (Sharma and Shrivastava, 2017). reported that blanching (100°C for 4 min) followed by solar cabinet drying (54 ± 5°C) with KMS (0.5%) contributed to a good rehydration ratio as well as good sensory acceptance of pointed gourd. Furthermore, the combination of carnauba wax (1:10), NaOCl (100 mg/L), and KMS (500 mg/L) under cold storage yielded the best result with respect to low physiological loss, high hue, low chroma, retained total soluble solids (TSS), and prolonged shelf-life of pointed gourd (Koley et al., 2009). Both packaging and chemical treatments in combination had a significant influence on the quality, color, ripening, and discard- or senescence-indicating parameters of avocado and pointed gourd fruits in terms of storage conditions (Koley et al., 2009; Sahoo et al., 2015; Sierra et al., 2019). All the postharvest treatments have been tested separately, and a concrete solution to the problems has yet to be reached. Only a few research works have been performed in combination with chemicals and packaging under ambient and refrigerated storage of pointed gourd.

Considering the abovementioned facts, it has been hypothesized that different packaging materials alone or in combination with user-friendly postharvest approaches could be effective to maintain pointed gourd acceptance for a certain period after storage. Thus, the present study was undertaken to find out the appropriate packaging and postharvest treatment for ambient and refrigerator storage of pointed gourd.

## Materials and methods

### Pointed gourd fruit sample

Pointed gourd (*Trichosanthes dioica* Roxb.) fruits of a local variety were harvested at the green stage of commercial maturity (20 days after pollination) from the research field of the Department of Horticulture, Bangabandhu Sheikh Mujibur Rahman Agricultural University (BSMRAU), Gazipur, Bangladesh, during summer season (March to August). The fruits were sorted into categories of uniform size, shape, and color, checked to be free from defects or blemishes, washed with tap water, and dried at room temperature for 15 min prior to treatment. The physiochemical and nutritional attributes were analyzed in the analytical laboratory of the Department of Horticulture, BSMRAU, while analyses of firmness and color contents were done at the Postharvest Technology Laboratory at Bangladesh Agricultural Research Institute (BARI), Joydevpur, Bangladesh.

### Packaging and postharvest treatment

The MAP treatment consisted of five different packaging materials of perforated and nonperforated polyethylene and polypropylene packets, brown paper bag, and control (without packaging). The polyethylene bags had a higher thickness of 25 µm compared to the polypropylene and brown paper bag of 20 µm thickness, with the same dimension of 2 m length × 15 cm width for each. In addition, four different postharvest management treatments (disinfectants and color retention) were used, namely, NaOCl (0.01% w/v), KMS (0.05% w/v), blanching at 100°C for 4 min, and control (without postharvest treatment).

### Experiment design

The experiment was designed into a two-factor randomized complete block design (factorial RCBD) with three replications, where the packaging was one factor having six levels and postharvest treatment was another one with four levels including control. The previously sorted air-dried fresh pointed gourd fruits were separated into two sample lots according to the two storage conditions of low temperature (LT, at 4°C) in the refrigerator and at room temperature (RT, at 30°C). The fruits from one lot were subjected to immersion in NaOCl (0.01% w/v) and KMS (0.05% w/v) (Sigma Aldrich, Germany) solution for 10 min following the procedure of (Haile, 2018) and (Koley et al., 2009) with some modifications. Blanching was done at 100°C temperature for 4 min using a hot water bath (Model: Memmert, WB-22, Germany) as mentioned by (Sharma and Shrivastava, 2017). The blanched samples were dipped in iced water at 0°C for 3 min to discharge the heat. The treated samples were completely dried with blotting paper prior to being weighed and packed. Thirty fruits were used in each postharvest treatment, and this process was repeated three times. Therefore, a total of 360 fruits were used for four types of postharvest treatment applications which were replicated three times. Half of the treated fruits were packed into the abovementioned five types of packaging (10 fruits at three packets each for a total of 150), and 30 treated fruits were kept open without packing as control prior to storage at low temperature (LT). The rest of the fruits of another lot were also processed similarly and kept at room temperature (RT). The treatment variables for packaging and postharvest treatment were tagged as P_1_ (control without packaging), P_2_ (perforated polythene, PPE with six pinholes of 0.3 mm of equal size), P_3_ (non-perforated polythene, NPE), P_4_ (perforated polypropylene, PPP with six pinholes of 0.3 mm of equal size), P_5_ (non-perforated polypropylene, NPP), P_6_ (brown paper bag, BP), C_1_ (control without chemical), C_2_ (NaOCl at 0.01% w/v), C_3_ (KMS at 0.05% w/v), and C_4_ (blanching at 100°C for 4 min), and the total number of treatment combinations for each replication was 24. Three replicate packets with treated fruits and control (without packaging and treatment) samples were stored in the refrigerator (LT, 4°C, 45% RH) and at ambient room temperature (RT, 30 ± 2°C, 65–75% RH) for further analyses. The pointed gourd fruits from each of the 24 treatments stored at LT and RT were analyzed to determine changes in physiochemical, nutrition, and color attributes at the end of the shelf-life of the respective treatments.

### Physiochemical analyses

#### Shelf-life

The shelf-life of the pointed gourd was estimated as the total duration starting after harvest to the endpoint of the remaining marketable quality. Therefore, regular monitoring was accomplished to find out the specific days required for losing the marketable quality of the postharvest treated pointed gourd at LT and RT conditions. The marketable quality of fruits was assessed by following the procedure of (Workneh et al., 2011) with some modifications. The descriptive quality attributes representing physical appearance were assessed subjectively by observing the level of glossiness, shriveling, smoothness, and visible decay with a panel of judges consisting of 20 respondents. Each respondent scored the studied fruit sample as per the following 1–5 rating scale: 1 = unusable, 2 = usable, 3 = fair, 4 = good, 5 = excellent. Fruits receiving a cumulative rating of 3 and above were considered marketable, and the total duration up to which a treated fruit sample could maintain its marketable acceptance score was expressed as its shelf-life in terms of the unit day.

#### Firmness

Firmness was measured using a TA-ST2i texture analyzer (Stable Micro System, Ltd., Godalming, Surrey, UK) with a 50-kg-load cell equipped with a 5-mm-diameter cylindrical probe. The test speed was 0.5 mm/s, the pretest speed was 10 mm/s, the probe reserving speed was 10 mm/s, the trigger force was 15 g, and the distance traveled by the probe inside the sample was 1 mm. Pointed gourds taken from each treatment were placed under the probe prior to force application. Firmness data was recorded at three places of each fruit, and an average was derived. Hardness was defined as a maximum force during compression and is expressed in kgf/cm^2^.

#### Physiological weight loss (%)

Weight loss was determined by weighing each sample before and after storage (at the end of the shelf-life) and expressed as the percent of weight loss per initial fruit weight. The results were denoted as the average of three replicates. The samples were weighed with the help of an electronic balance having 0.0001 g as the tiniest count by the following formula:


$$\text{Physiological weight loss }\left(\%\right)=\left(\frac{\text{Initial weight}-\text{final weight}}{\text{Initial weight}}\;\times 100\right)$$

#### Total soluble solids

Samples from each type of treatment were homogenized in a grinder and centrifuged for 20 min at 5,000 rpm. The supernatant phase was used for the estimation of TSS as suggested by (Hassan et al., 2022) and expressed as a percentage.

#### Chlorophyll content

Chlorophyll was extracted from the fruit sample using the standard operating procedure described by the Scientific Engineering Response and Analytical Services, Environmental Protection Agency of the United States (SERAS 1994) and the procedure adopted by Ku et al. (Ku et al., 2013) and Rajalakshmi and Bano (2015). Briefly, the procedure was to take 2 g of a fruit, cut into small pieces, and homogenize with 2 ml of 0.1 N NH_4_OH extraction solutions to form a fine slurry. The plant material that adhered to the pestle was washed off using 3 ml of extraction solution, and then 5 ml of 80% aqueous acetone solution was added. The sample extracts were centrifuged at 3,000 rpm for 10 min. The supernatant solution was decanted, and the final volume was brought up to 10 ml using 80% aqueous acetone. The extracted sample was then analyzed using a spectrophotometer (APEL, UV-VIS Spectrophotometer, PD – 303 UV, PD 33-3-OMS-101 b, Japan) operating at wavelengths of 645 and 663 nm. Initially, aqueous acetone 80% was used as a blank to zero the spectrophotometer. The equations reported by (Arnon, 1949) were then used to calculate the levels of chlorophyll *a*, chlorophyll *b*, and total chlorophyll.


$$\text{Chlorophyll }a\;\left(µ\text{g}/\text{g}\right)\;=\;20.2\;\left(A_{645}\right)\;+\;8.02\;\left(A_{663}\right)$$

Chlorophyll b (µg/g)= 12.7 (A663)− 2.69 (A645)

Total chlorophyll (µg/g)=(20.2  A645 + 8.20  A663)  DF

where *A*_645_ = absorbance at a wavelength of 645 nm; *A*_663_ = absorbance at a wavelength of 663 nm; 20.2, 8.02, 12.7, and 2.69 = absorbance coefficients; DF = dilution factor $\;\frac{V}{1000\;\times \;W}$ =; = final volume of the extract in 80% acetone, and *W* = fresh weight of tissue.

#### Color

The color of the pointed gourd samples at different storage conditions was detected using Hunter Lab Colorimeter (LabScan XE, Hunter Lab Colorimeter, DP-9000D25A, Reston, USA). Colorimetric data was taken from both sides of the fruit after calibration with a black and white standard tile. The results were expressed in terms of L*, a*, and b* values, where L* represents luminosity or lightness (where 0 to 100 represents black→white), a* represents chromaticity on a green (-) to red (+) axis, and b* represents chromaticity on a blue (−b) to yellow (+b) axis. The following equation was used to compute the hue angle (h*) and chroma (C*), two color characteristics commonly employed for green vegetables as mentioned by (Pathare et al., 2012).

hue angle (h*) = $tan^{-1}\left(\frac{b*}{a*}\right)$

     chroma (C*) = $\sqrt{{\text{a*}}^{2}+{\text{b*}}^{2}}$

#### Nutrition analyses

#### Ascorbic acid

Ascorbic acid was determined according to the method proposed by Pleshkov (1976). Briefly, 10 g of the studied fruit sample was blended and homogenized with 50 ml of distilled water. The ground sample was centrifuged at 4,000 rpm for 20 min, and the supernatant (10 ml) was used to determine ascorbic acid following the titration method. The titration sample was prepared by adding 5 ml of 5% potassium iodide, 2 ml of 2% starch solution, and 2 ml of 100% glacial acetic acid to the supernatant. Finally, it was titrated with 0.001 N KIO_3_ solution. Afterward, total ascorbic acid was calculated using the following formula:


$$A\text{scorbic acid content}\;\left(\frac{\text{mg}}{\text{l}00}\text{g}\right)=\frac{F\;\times \;V1\;\times \;V2\;\times 100}{V3\times \;W}$$

Where *F* = 0.088 mg of ascorbic acid per ml of 0.001 N KIO_3_, V1 = titrated volume of KIO_3_ ml, V2 = total volume of the sample extract (ml), V3 = volume of the extract (ml) taken for titration, and *w* = weight of the sample taken (g).

#### β-carotene

β-carotene pigments (known as naturally occurring provitamin-A carotenoids) were extracted by 80% acetone and estimated following the methods of Hasan et al. (Hassan et al., 2022). Initially, 2 g of pointed gourd fruit pulp was taken in a flask, and 20 ml of acetone–hexane (4:6) solution was mixed homogeneously. The sample was centrifuged, and the optical density of the supernatant was measured using a double-beam spectrophotometer (model: APEL, UV-VIS Spectrophotometer, PD-303 UV, PD 33-3-OMS-101 b, Japan) at 663, 645, 505, and 453 nm. The amount of β-carotene was determined using the following formula (mg/100 g on a weight basis):


. β−carotene = 0.216 (OD663)+ 0.452 (OD453)− 1.22 (OD645)− 0.304 (OD505)

where OD663 = optical density at 663-nm wavelength, OD453 = optical density at 453-nm wavelength, OD645 = optical density at 645-nm wavelength, and OD505 = optical density at 505-nm wavelength; 0.216, 0.452, 1.22, and 0.304 = absorption coefficient of the respective absorbance value.

### Statistical analyses

All data were expressed as the means ± standard deviations of the triplicate measurements (Azad et al., 2022; Azam et al., 2022; Prodhan et al., 2022; Rahman et al., 2022). Two-way analysis of variance (ANOVA) was done to compute differences between mean values considering the effect of packaging materials and postharvest treatments. *Post-hoc* multiple comparisons (Tukey HSD test) were carried out to test significant differences among treatment means at a level of *P<*0.05. Correlation matrix and cluster analysis were performed to sort out the interrelationship among the studied color and nutritional compositions of pointed gourd fruits. Afterward, principal component analysis (PCA) was performed to show the patterns of all the estimated correlated color and nutritional properties of the pointed gourd sample in the reduced dimensions of newly obtained factors; those were denoted as Dim1 (Dimension1 or PC1) and Dim2 (Dimension2 or PC2). The number of new variables was chosen based on the eigenvalues, which represent the total amount of variance that can be explained by a given principal component. In this approach, any component (factor) with an eigenvalue >1.00 was considered for interpretation focusing on the factor loadings of the independent variables (packing materials and postharvest treatment) and the contribution of each of the studied dependent variables (color and nutritional composition) to the total variations. The factor loadings and the contributions of each of the studied dependent variables determined using different packages (Agricole, facatominer, factoextra, ggplot2, and corrplot) of R program (version 4.0.2).

## Results and discussion

### Physiochemical attributes

#### Shelf-life

Fruits packed in a non-perforated polyethylene bag (NPE) with KMS treatments were able to maintain a good physical appearance for the longest periods (23 and 10 days) both at LT and RT storage conditions, respectively (**Table 1**). However, perforated and non-perforated packing without any chemical or blanching turned out as the second best (20 and 7 days), while non-perforated packing (NPE and NPP) in combination with either NaOCl or KMS resulted as the third best (17 and 5 to 6 days, respectively) performance with respect to shelf-life at LT and RT storage. The present results revealed that non-perforated packaging material in combination with KMS treatment performed well in extending the shelf-life of pointed gourd both at LT and RT. It might have happened due to the modified atmosphere condition prevailing with higher CO_2_ and lower O_2_ in the non-perforated PP and PE, causing lower respiration and leading to extended shelf-life (Mahajan et al., 2006; Maftoonazad and Ramaswamy, 2008). Meanwhile, among the two storage conditions, LT sharply performed the best in NPE and NPP packaging conditions regarding prolonged shelf-life. It is because having low temperature and light conditions inside the refrigerator controls O_2_ depletion and increase in CO_2_ to reduce the respiration of the stored products (Sharma and Shrivastava, 2017). In addition, among the postharvest treatments, KMS responded well in maximizing fruit acceptability after reducing the enzyme activity related to respiration and decay (Liu et al., 2007; Rojas-Graü et al., 2009). The results were concurrent with the findings of (Jafri et al., 2013) who stated that a higher shelf-life of mushrooms was observed from MAP with chemical treatment in storage.

**Table 1:** Physiological attributes of the pointed gourd up to acceptance level under different postharvest treatment and packaging conditions.

Treatment	Shelf-life (days)		Firmness (kgf/cm^2^)		Weight loss (%)		TSS (%)	
	LT (4°C)	RT (30°C)	LT (4°C)	RT (30°C)	LT (4°C)	RT (30°C)	LT (4°C)	RT (30°C)
P_1_C_1_	3 ± 0.0e	2 ± 0.0d	0.14 ± 0.01a–c	0.10 ± 0.01c–f	20.0 ± 0.2b	31.1 ± 1.4a	9.6 ± 0.1ab	9.1 ± 0.1c–g
P_1_C_2_	3 ± 0.0e	2 ± 0.0d	0.14 ± 0.01a–c	0.11 ± 0.01b–f	12.0 ± 0.4cd	22.9 ± 1.7cd	8.7 ± 0.2fg	9.9 ± 0.1ab
P_1_C_3_	3 ± 0.0e	2 ± 0.0d	0.11 ± 0.01bc	0.10 ± 0.01c–f	11.3 ± 0.6d	16.7 ± 1.6e–h	9.4 ± 0.2bc	9.1 ± 0.1c–g
P_1_C_4_	1 ± 0.0e	1 ± 0.0d	0.16 ± 0.01ab	0.11 ± 0.01b–f	8.7 ± 1.1ef	20.7 ± 1.6c–e	9.3 ± 0.3b–d	9.4 ± 0.2b–e
P_2_C_1_	10 ± 0.0d	7 ± 0.6b	0.16 ± 0.00ab	0.13 ± 0.01a–f	6.5 ± 0.28gh	24.6 ± 1.5bc	9.1 ± 0.1c–e	9.2 ± 0.3c–f
P_2_C_2_	10 ± 0.0d	6 ± 2.1bc	0 ± 0.00d	0.10 ± 0.03c–f	7.2 ± 0.5g	14.5 ± 1.0gh	8.9 ± 0.4d–g	9.2 ± 0.3c–f
P_2_C_3_	15 ± 0.0c	5 ± 0.0c	0 ± 0.00d	0.07 ± 0.07f	12.3 ± 1.1cd	12.9 ± 0.7h	8.5 ± 0.0g–i	9.6 ± 0.1b–d
P_2_C_4_	1 ± 0.0e	1 ± 0.0d	0.17 ± 0.01ab	0.14 ± 0.00a–e	1.5 ± 0.4kl	3.4 ± 0.4ij	8.5 ± 0.0g–i	9.0 ± 0.0d–g
P_3_C_1_	20 ± 0.0b	7 ± 0.6b	0.17 ± 0.02a	0.16 ± 0.02a–c	0.9 ± 0.1kl	1.4 ± 0.4j	10.0 ± 0.1a	8.4 ± 0.1g
P_3_C_2_	17 ± 2.9c	5 ± 0.0c	0.16 ± 0.05ab	0.09 ± 0.09ef	0.1 ± 0.0l	2.7 ± 0.1ij	9.0 ± 0.0c–f	8.8 ± 0.1e–g
P_3_C_3_	23 ± 2.9a	10 ± 0.0a	0.19 ± 0.01a	0.15 ± 0.00a–d	3.1 ± 0.0j	3.5 ± 0.2ij	9.2 ± 0.0b–d	9.8 ± 0.3a–c
P_3_C_4_	1 ± 0.0e	1 ± 0.0d	0.18 ± 0.00a	0.17 ± 0.01ab	0.6 ± 0.1kl	0.7 ± 0.2j	8.2 ± 0.2i	8.8 ± 0.3e–g
P_4_C_1_	10 ± 0.0d	7 ± 0.0b	0.15 ± 0.02ab	0.11 ± 0.01b–f	8.6 ± 0.2ef	28.0 ± 2.7ab	9.4 ± 0.2bc	9.0 ± 0.3d–g
P_4_C_2_	10 ± 0.0d	6 ± 2.1bc	0.08 ± 0.09c	0.12 ± 0.03a–f	19.2 ± 1.1b	28.8 ± 10.3ab	9.3 ± 0.1bc	9.5 ± 0.0b–d
P_4_C_3_	10 ± 0.0d	5 ± 0.0c	0 ± 0.00d	0.08 ± 0.08f	12.9 ± 0.8c	16.6 ± 1.5e–h	8.3 ± 0.8hi	9.8 ± 0.3a–c
P_4_C_4_	1.0 ± 0.0e	1 ± 0.0d	0.17 ± 0.01ab	0.15 ± 0.01a–d	1.5 ± 0.3kl	6.3 ± 0.4i	8.5 ± 0.0g–i	9.5 ± 0.5b–d
P_5_C_1_	15 ± 0.0c	7 ± 2.0b	0.17 ± 0.02ab	0.09 ± 0.08d–f	1.8 ± 0.1jk	1.9 ± 0.1ij	9.3 ± 0.3b–d	8.5 ± 0.5fg
P_5_C_2_	17 ± 2.9c	6 ± 1.5b	0.16 ± 0.05ab	0.18 ± 0.02a	5.8 ± 0.8hi	19.0 ± 2.4d–g	9.0 ± 0.0c–f	9.1 ± 0.1c–g
P_5_C_3_	17 ± 2.9c	7 ± 0.6b	0.19 ± 0.01a	0.11 ± 0.01b–f	7.6 ± 0.3fg	4.4 ± 0.4ij	8.8 ± 0.3e–g	9.3 ± 0.2b–e
P_5_C_4_	1 ± 0.0e	1 ± 0.0d	0.18 ± 0.01a	0.16 ± 0.01a–c	0.6 ± 0.2kl	0.8 ± 0.1j	8.2 ± 0.2i	8.7 ± 0.4e–g
P_6_C_1_	3 ± 0.0e	2 ± 0.0d	0.19 ± 0.02a	0.11 ± 0.01b–f	12.4 ± 2.3cd	30.8 ± 0.6a	8.6 ± 0.1gh	10.5 ± 0.3a
P_6_C_2_	3 ± 0.0e	2 ± 0.0d	0.03 ± 0.06d	0.15 ± 0.05a–d	23.8 ± 1.5a	19.6 ± 2.6d–f	9.1 ± 0.1c–e	9.6 ± 0.1b–d
P_6_C_3_	3 ± 0.0e	2 ± 0.0d	0.16 ± 0.02ab	0.11 ± 0.01b–f	9.7 ± 0.5e	13.1 ± 1.3h	8.5 ± 0.0g–i	9.6 ± 0.1b–d
P_6_C_4_	1 ± 0.0e	1 ± 0.0d	0.14 ± 0.00a–c	0.10 ± 0.00d–f	5.1 ± 0.9i	15.8 ± 2.5f–h	8.6 ± 0.1gh	9.5 ± 0.0b–d
LS	**	**	*	*	**	**	**	**

Numeric values (±) are standard deviation, and letters in the same row (a–l) indicate a significant difference.

P_1_ and C_1_, control; P_2_ and P_3_, perforated and non-perforated polyethylene (PPE and NPE, respectively); P_4_ and P_5_, perforated and non-perforated polypropylene (PPP and NPP, respectively); P_6_, brown paper (BP); C_2_, NaOCl; C_3_, KMS; C_4_, blanching.

*p < 0.05, **p < 0.01, ***p < 0.001. *** as p<0.001 that means probability of significance level at 0.1%

#### Firmness

The firmness of the fresh sample was 0.24 kgf/cm^2^, and it was reduced dramatically with the increase in storage duration and temperature. Thus, blanching had higher firmness but for the shortest period of storage time (1 day) (**Table 1**). Besides this, the highest firmness was recorded from non-perforated polybags (NPE and NPP) with KMS (0.19 kgf/cm^2^) at the LT condition. Meanwhile, at RT condition, the best result was obtained from NPP with NaOCl (0.18 kgf/cm^2^) which was at par with NPE with no chemical and KMS treatment (0.16 and 0.15 kgf/cm^2^) (**Table 1**). Tan et al. (Tan et al., 1999) also reported a higher firmness reduction in bitter gourd at ambient storage without treatment. The trend of decrease in firmness during the storage might be due to loss of moisture by transpiration and respiration as well as conversion of organic and inorganic metabolites. As a consequence, the middle lamella of the cell wall, its strength, and degraded cell-to-cell bonding led to cellular swell as well as reducing the puncture force of firmness (Arnon et al., 2014; Sogvar et al., 2016).

#### Physiological loss in weight

The physiological loss in weight (PLW%) was noticeably enhanced with the extension of the storage period for all the treatments after storage at LT and RT (**Table 1**). The PLW% was found maximum in control (31.1%), which was statistically similar with BP without chemicals (30.8%) at RT, and a similar trend of increasing PLW% was observed in BP with NaOCl (23.8%), followed by control (20%) at LT. In contrast, the minimum PLW% was noticed in the fruits packed in NPE and NPP treated with blanched and KMS at RT, while only 0.1% PLW was recorded in NPE-packed fruits in combination with NaOCl at LT. This minimal loss in the NPE and NPP fruits might be due to the retardation in the process of respiration- and transpiration-related metabolic activities during storage (Tirkey et al., 2014). This finding was concurrent with the previous reports of (Liu et al., 2007) and (Sahoo et al., 2015) who found lower physiological loss from NPE and NPP under refrigerated storage of tomato and pointed gourd. Waghmare et al. (Waghmare and Annapure, 2013) indicated the acceptable texture of fresh-cut papaya up to 25 days with MAP and chemical treatment combination (CaCl_2_ 1% and citric acid 2% w/v).

#### Total soluble solid

TSS content is one of the most important indicators that determine the quality of pointed gourd. Therefore, the slower conversion of complex carbohydrates to TSS is desirable. Blanched pointed gourd packed with a different packaging material had lower TSS values due to its shortest storage life (1 day). Apart from blanching data, the perforated wrapping (PPP and PPE) along with KMS treatment was retained (initial TSS 7.5%), and there was more TSS (8.3 and 8.5%, respectively) at LT compared to the initial content (7.5%). Kalra et al. (Kalra et al., 1983) also recorded a lower TSS value from KMS-treated bitter gourd when stored at a cold temperature. On the contrary, the TSS value was the highest (9.6%) in the control treatment (P_1_C_1_) under the LT condition. On the other hand, at RT, a lower TSS reduction was observed in non-perforated NPE (8.4%), which was statistically identical to non-perforated NPP (8.5%) followed by NaOCl (8.8%). The results revealed that fruits packed in both perforated and non-perforated PE and PP could control the gradual reduction of carbohydrates to TSS during storage at LT and RT, whereas in control samples at the storage condition of LT and RT, the TSS conversion rate was higher, which was due to the faster metabolic activities that went through respiration (Niyomlao et al., 2000; Sahoo et al., 2015). Meanwhile, brown paper (P6) without chemical (C_1_) treatment was not able to check the TSS conversion at RT (**Table 1**) (Koley et al., 2009). also recorded a higher TSS value from no chemical and lower TSS from wax + NaOCl + KMS-treated pointed gourd. The present findings were substantiated by Waghmare et al. (Waghmare and Annapure, 2013) who suggested that a combination of chemical and packaging treatment would be effective to check the TSS conversion in papaya.

#### Total chlorophyll

The study findings revealed that postharvest treatments and packaging materials were able to reduce the total chlorophyll (5.20 mg/g) breakdown at both the storage conditions of LT and RT. As observed, brown paper without chemicals retained the maximum total chlorophyll (2.50 mg/g) that was identical to NPE without chemicals (1.28 mg/g) and perforated bagging (PPE and PPP) with NaOCl (1.39 mg/g and 1.52 mg/g, respectively) at LT storage condition. On the other side, NPP with no chemical preserved the maximum total chlorophyll (2.37 mg/g), and the rest were identical at RT condition (**Table 2**). It was evident that packaging with a disinfectant chemical under low temperature limited the conversion of chlorophyll into pheophytin (Jeong et al., 2003; Maftoonazad and Ramaswamy, 2005) and the activity of pectin methylesterase (Olivas and Barbosa-Canovas, 2005), which interrupts xanthophyll and anthocyanin dominance and β-carotene formation as well as yellowing (Sahoo et al., 2015). Hence, in the present study, fruits treated with KMS and NaOCl retained a higher chlorophyll level in the range of 0.45–0.80 and 0.68–0.84, respectively, irrespective of packaging materials at LT and RT, leading to extending the shelf-life of pointed gourd.

**Table 2:** Pigments of pointed gourd up to acceptance level under different postharvest treatment and packaging conditions.

Treatment	Chlorophyll *a* (mg/g)		Chlorophyll *b* (mg/g)		Total chlorophyll (mg/g)	
	LT (4°C)	RT (30°C)	LT (4°C)	RT (30°C)	LT (4°C)	RT (30°C)
P_1_C_1_	0.34 ± 0.05bcd	0.39 ± 0.06b	0.17 ± 0.01b	0.18 ± 0.04b	0.51 ± 0.04b	0.58 ± 0.01b
P_1_C_2_	0.48 ± 0.08a–d	0.47 ± 0.16b	0.23 ± 0.08b	0.24 ± 0.05b	0.71 ± 0.16b	0.71 ± 0.22b
P_1_C_3_	0.38 ± 0.18a–d	0.53 ± 0.09ab	0.17 ± 0.07b	0.28 ± 0.02b	0.55 ± 0.25b	0.80 ± 0.11b
P_1_C_4_	0.33 ± 0.01bcd	0.45 ± 0.19b	0.07 ± 0.04b	0.09 ± 0.05b	0.41 ± 0.05b	0.54 ± 0.24b
P_2_C_1_	0.44 ± 0.00a–d	0.36 ± 0.10b	0.21 ± 0.02b	0.20 ± 0.07b	0.65 ± 0.02b	0.53 ± 0.15b
P_2_C_2_	0.89 ± 0.49ab	0.32 ± 0.20b	0.50 ± 0.26b	0.20 ± 0.16b	1.39 ± 0.75ab	0.53 ± 0.36b
P_2_C_3_	0.11 ± 0.01d	0.27 ± 0.02b	0.09 ± 0.01b	0.18 ± 0.01b	0.19 ± 0.02b	0.44 ± 0.02b
P_2_C_4_	0.35 ± 0.08bcd	0.51 ± 0.27b	0.10 ± 0.10b	0.10 ± 0.09b	0.44 ± 0.18b	0.61 ± 0.36b
P_3_C_1_	0.83 ± 0.02abc	0.38 ± 0.14b	0.45 ± 0.06b	0.25 ± 0.15b	1.28 ± 0.08ab	0.61 ± 0.27b
P_3_C_2_	0.38 ± 0.22a–d	0.55 ± 0.06ab	0.19 ± 0.09b	0.29 ± 0.05b	0.58 ± 0.31b	0.84 ± 0.12b
P_3_C_3_	0.63 ± 0.23a–d	0.44 ± 0.11b	0.37 ± 0.05b	0.25 ± 0.03b	1.00 ± 0.28b	0.69 ± 0.13b
P_3_C_4_	0.41 ± 0.00a–d	0.28 ± 0.07b	0.12 ± 0.07b	0.05 ± 0.04b	0.53 ± 0.07b	0.31 ± 0.12b
P_4_C_1_	0.61 ± 0.02a–d	0.48 ± 0.08b	0.25 ± 0.03b	0.28 ± 0.06b	0.86 ± 0.05b	0.77 ± 0.16b
P_4_C_2_	0.71 ± 0.48a–d	0.34 ± 0.25b	0.81 ± 0.69ab	0.22 ± 0.19b	1.52 ± 1.18ab	0.56 ± 0.48b
P_4_C_3_	0.65 ± 0.04a–d	0.26 ± 0.01b	0.33 ± 0.00b	0.19 ± 0.02b	0.97 ± 0.04b	0.45 ± 0.00b
P_4_C_4_	0.41 ± 0.21a–d	0.27 ± 0.05b	0.13 ± 0.12b	0.05 ± 0.04b	0.53 ± 0.34b	0.29 ± 0.12b
P_5_C_1_	0.28 ± 0.00bcd	1.13 ± 0.66a	0.17 ± 0.02b	1.24 ± 0.96a	0.44 ± 0.02b	2.37 ± 1.62a
P_5_C_2_	0.55 ± 0.33a–d	0.50 ± 0.12b	0.24 ± 0.09b	0.26 ± 0.07b	0.78 ± 0.41b	0.76 ± 0.14b
P_5_C_3_	0.64 ± 0.24a–d	0.41 ± 0.01b	0.29 ± 0.09b	0.26 ± 0.00b	0.93 ± 0.33b	0.68 ± 0.00b
P_5_C_4_	0.40 ± 0.08a–d	0.41 ± 0.13b	0.17 ± 0.11b	0.12 ± 0.12b	0.57 ± 0.20b	0.52 ± 0.25b
P_6_C_1_	0.21 ± 0.08cd	0.59 ± 0.31ab	0.10 ± 0.04b	0.60 ± 0.45ab	0.31 ± 0.12b	1.19 ± 0.75b
P_6_C_2_	0.33 ± 0.12bcd	0.44 ± 0.04b	0.15 ± 0.01b	0.24 ± 0.03b	0.47 ± 0.12b	0.68 ± 0.00b
P_6_C_3_	1.04 ± 0.64a	0.37 ± 0.02b	1.46 ± 1.19a	0.14 ± 0.02b	2.50 ± 1.83a	0.51 ± 0.00b
P_6_C_4_	0.27 ± 0.04bcd	0.41 ± 0.11b	0.09 ± 0.04b	0.07 ± 0.06b	0.36 ± 0.07b	0.48 ± 0.17b
LS	***	*	***	*	***	*

Numeric values (±) are standard deviation, and letters in the same row are significant differences.

P_1_ and C_1_, control; P_2_ and P_3_, perforated and non-perforated polyethylene (PPE and NPE, respectively); P_4_ and P_5_, perforated and non-perforated polypropylene (PPP and NPP, respectively); P_6_, brown paper (BP); C_2_, NaOCl; C_3_, KMS; C_4_, blanching.

*p < 0.05, **p < 0.01, ***p < 0.001.

#### Color

Since surface color is the first and most critical quality parameter judged by the consumer at the time of purchase, different color indices may require getting a detailed characterization of the quality attributes of fresh commodities even after storing under different conditions. In this study, color measurements on different packaged pointed gourds treated with postharvest treatments were monitored under RT (ambient) and LT (cold storage) conditions throughout their shelf-life (**Table 3**). The results indicated that the L* (lightness) values of wrapped and unwrapped pointed gourd decrease with an increase in storage period under both storage conditions (RT and LT). Meanwhile, there was a consistent decrease in the greenness value (-a*) and an increase in the yellowness value (+b*) for both storage samples irrespective of whether they are wrapped or unwrapped (**Table 3**). However, we calculated the hue (h*) and chroma (C*) values (using a* and b* values) to get a clear understanding of the greenness or yellowness of pointed gourd as the fruits are green in fresh conditions.

**Table 3:** Commission Internationale de I’Eclairage color parameters of pointed gourd up to acceptance level under different postharvest treatment and packaging conditions.

Treatment	CIE lightness coordinate (L*)		a*		b*		Hue value (h*)		Chroma (C*)	
	LT (4°C)	RT (30°C)	LT (4°C)	RT (30°C)	LT (4°C)	RT (30°C)	LT (4°C)	RT (30°C)	LT (4°C)	RT (30°C)
P_1_C_1_	39.1 ± 0.4f–i	74.1 ± 4.5	-7.2 ± 0.0a–d	-7.6 ± 0.1ab	17.6 ± 1.5g	20.6 ± 0.2h	19.4 ± 1.2ij	33.9 ± 2.1l	69.8 ± 0.2g	21.9 ± 0.1i
P_1_C_2_	35.2 ± 4.3hi	71.8 ± 1.8	-7.1 ± 0.2a–d	-4.9 ± 0.7de	21.5 ± 2.8fg	34.9 ± 0.3abc	23.0 ± 2.7g–j	51.3 ± 0.4a–d	81.8 ± 1.1a–c	35.4 ± 0.2bc
P_1_C_3_	44.1 ± 2.2c–g	72.8 ± 0.3	-7.7 ± 0.1a–d	-8.4 ± 0.6ab	24.8 ± 0.7d–g	24.2 ± 1.3fgh	25.8 ± 0.6e–g	44.2 ± 0.8e–j	71.3 ± 0.5fg	25.2 ± 1.1e–i
P_1_C_4_	46.7 ± 0.4b–f	85.1 ± 0.6	-2.2 ± 0.2h	-1.5 ± 0.3h	26.4 ± 4.3c–g	22.8 ± 0.2gh	25.2 ± 3.7f–i	40.0 ± 0.1h–l	85.9 ± 0.2a	23.0 ± 0.1hi
P_2_C_1_	46.2 ± 0.4b–f	76.2 ± 1.1	-8.3 ± 0.9abc	-6.9 ± 1.1bc	34.4 ± 0.3a–d	27.0 ± 2.6c–h	35.5 ± 0.4a–d	45.1 ± 1.7e–h	77.8 ± 2.9cd	28.8 ± 2.3de
P_2_C_2_	49.0 ± 0.6a–e	76.1 ± 0.9	-7.7 ± 0.4a–d	-3.5 ± 0.3efg	31.9 ± 0.9a–e	42.6 ± 0.2a	32.6 ± 0.6cd	56.4 ± 0.8a	85.0 ± 0.7a	42.8 ± 0.2a
P_2_C_3_	51.8 ± 1.4a–c	80.8 ± 0.0	-6.3 ± 0.1a–e	-8.8 ± 0.5a	38.7 ± 0.8ab	28.0 ± 2.2c–h	38.9 ± 0.7ab	46.8 ± 1.1d–g	71.9 ± 2.1fg	28.7 ± 1.5d–f
P_2_C_4_	34.7 ± 1.9i	83.0 ± 0.0	-2.4 ± 0.2h	-3.3 ± 0.5efg	19.0 ± 0.5g	24.9 ± 0.3e–h	19.3 ± 0.5ij	38.6 ± 0.5i–l	82.7 ± 1.0ab	25.3 ± 0.0e–i
P_3_C_1_	52.9 ± 0.7ab	77.8 ± 2.7	-8.0 ± 1.6abc	-7.6 ± 0.8ab	40.2 ± 2.6a	32.9 ± 1.2cde	40.4 ± 1.9a	53.0 ± 5.2a–c	77.0 ± 1.9c–e	33.8 ± 1.0c
P_3_C_2_	51.5 ± 1.2a–d	63.6 ± 23.7	-7.2 ± 2.4a–d	-7.2 ± 0.5abc	35.5 ± 2.0abc	34.0 ± 1.0bcd	35.9 ± 1.9a–d	47.8 ± 0.9c–f	77.9 ± 0.3b–d	35.0 ± 1.0bc
P_3_C_3_	50.5 ± 7.0a–e	81.1 ± 2.4	-5.7 ± 1.3c–f	-8.1 ± 0.7ab	41.3 ± 4.5a	26.9 ± 0.6c–h	39.6 ± 4.7ab	43.6 ± 3.0f–j	72.7 ± 1.4e–g	28.1 ± 0.3e–g
P_3_C_4_	42.6 ± 0.5e–h	80.5 ± 0.2	-3.3 ± 0.2fgh	-2.8 ± 0.2fgh	19.5 ± 0.4g	24.2 ± 0.7fgh	19.7 ± 0.4h–i	37.2 ± 0.4kl	83.6 ± 0.3a	24.4 ± 0.7g–i
P_4_C_1_	43.9 ± 1.8c–g	73.9 ± 1.3	-8.5 ± 0.1ab	-7.7 ± 0.6ab	30.5 ± 2.4b–f	32.0 ± 1.5c–f	31.0 ± 2.0d–f	52.3 ± 2.0a–d	77.4 ± 3.0c–e	34.2 ± 3.1c
P_4_C_2_	42.8 ± 4.0e–h	76.2 ± 2.3	-7.4 ± 0.4a–d	-4.0 ± 0.4def	32.1 ± 4.3a–e	30.5 ± 8.4c–g	31.8 ± 3.5c–e	44.6 ± 3.8e–i	83.3 ± 2.0a	24.6 ± 1.0f–i
P_4_C_3_	48.3 ± 3.6a–e	77.7 ± 6.1	-5.9 ± 2.5b–f	-7.8 ± 0.2ab	35.7 ± 8.3abc	31.6 ± 1.1c–f	37.4 ± 3.2a–c	54.4 ± 0.7ab	76.0 ± 0.1d–f	32.2 ± 0.9cd
P_4_C_4_	36.3 ± 0.5g–i	81.8 ± 0.0	-2.7 ± 0.2gh	-1.8 ± 0.1gh	17.7 ± 0.1g	24.0 ± 0.1fgh	17.9 ± 0.1j	41.1 ± 0.1g–k	85.7 ± 0.1a	24.1 ± 0.15g–i
P_5_C_1_	50.8 ± 1.1a–d	80.5 ± 2.5	-5.2 ± 0.1d–g	-7.5 ± 0.6ab	36.8 ± 0.5ab	30.1 ± 3.0c–g	37.4 ± 0.4a–c	53.5 ± 4.3a–c	77.0 ± 3.8c–e	35.2 ± 2.5bc
P_5_C_2_	49.0 ± 2.9a–e	63.6 ± 23.1	-7.7 ± 2.0a–d	-4.5 ± 0.4def	34.8 ± 0.3abc	41.5 ± 0.5ab	36.5 ± 1.5a–d	54.0 ± 0.7ab	85.5 ± 2.2a	42.3 ± 0.9a
P_5_C_3_	56.1 ± 2.2a	77.5 ± 1.8	-8.9 ± 0.9a	-5.5 ± 1.6cd	36.3 ± 8.6ab	34.1 ± 7.1bcd	33.6 ± 2.0b–d	52.2 ± 2.1a–d	84.8 ± 1.6a	38.9 ± 2.3ab
P_5_C_4_	35.1 ± 0.1hi	82.7 ± 0.4	-2.6 ± 0.3gh	-1.8 ± 0.1gh	19.3 ± 0.3g	26.5 ± 0.1d–h	19.3 ± 0.1ij	38.2 ± 0.2j–l	85.9 ± 0.1a	26.5 ± 0.1e–h
P_6_C_1_	43.8 ± 0.6d–g	70.6 ± 0.1	-8.2 ± 0.2abc	-6.8 ± 0.7bc	23.3 ± 0.7efg	27.9 ± 0.1c–h	24.9 ± 0.7g–i	43.3 ± 1.1f–j	75.9 ± 1.2d–f	28.8 ± 0.2de
P_6_C_2_	40.0 ± 4.4f–i	72.1 ± 0.1	-7.6 ± 0.4a–d	-6.8 ± 0.1bc	24.1 ± 0.8efg	31.4 ± 1.4c–f	25.5 ± 0.8f–h	50.1 ± 0.6b–e	78.1 ± 0.4b–d	32.7 ± 1.0cd
P_6_C_3_	39.5 ± 2.0f–i	72.0 ± 0.3	-8.2 ± 0.5abc	-7.6 ± 0.5ab	25.0 ± 1.3d–g	27.2 ± 0.6c–h	25.8 ± 1.0e–g	48.4 ± 0.9b–f	74.6 ± 0.6d–g	27.9 ± 0.4e–g
P_6_C_4_	37.1 ± 0.6g–i	81.2 ± 0.7	-3.6 ± 0.3e–h	-3.0 ± 0.2fgh	22.8 ± 0.6efg	27.5 ± 2.3c–h	22.8 ± 0.5g–j	40.8 ± 0.2g–k	83.8 ± 0.3a	27.0 ± 2.0e–h
LS	***	NS	***	***	***	***	***	***	***	***

Numeric values (±) are standard deviation, and letters in the same row (a–l) are significant differences.

P_1_ and C_1_, control; P_2_ and P_3_, perforated and non-perforated polyethylene (PPE and NPE, respectively); P_4_ and P_5_, perforated and non-perforated polypropylene (PPP and NPP, respectively); P_6_, brown paper (BP); C_2_, NaOCl; C_3_, KMS; C_4_, blanching. (***p < 0.00, NS, not significant).

The initial lightness (L*) of the fresh sample was 44.22, and the trend of increase in lightness was quicker at RT than in LT conditions. However, the L* value varied significantly according to packaging materials and postharvest treatment (**Table 3**). Lightness was the highest in NPP with KMS (56.1), wherein NPE with NaOCl and KMS were slightly lower but very close to higher values (51.5 and 50.1, respectively) (**Table 3**). This slightly diffused value may be due to physiological loss caused by respiration spreading moisture on the product surface and diffused luminosity (L*) leading to discoloration (Manjunatha and Anurag, 2014).

The greenness of the initial fresh sample was -71.1, and it was used to compare the greenness with the treated sample. It has been observed that the highest h* was calculated from NPE with no chemical (40.4), followed by NPE with KMS (39.6) and PPE with KMS (38.9) at LT storage conditions. On the contrary, treatment PPE with NaOCl (56.4) gave the highest h* in the RT condition. The lowest h* value was obtained from the control (33.9). The lightness (L*) and hue (h*) values were comparatively lower at LT storage than at RT.

The initial chroma value (C*) of the fresh sample was 26.9, and at the end of shelf-life it was higher at LT than RT storage conditions according to the different treatments (**Table 3**). The lowest C* value at LT storage condition was from control (69.8), which was statistically similar to PPE and NPE with KMS (71.9 and 72.7). On the other hand, the lowest C* value at RT was also computed from the control (21.9), and this was also similar to no packing with KMS (25.2), PPP with NaOCl (24.6), and NPE with KMS (28.1). Pathare et al. (Pathare et al., 2012) reviewed that color is an important sensory, nutritional, and consumer acceptability indicator, and the changes of white color to yellowish or light brown color or the development of yellowish or brownish skin color of pointed gourd could be due to the changing activities of endogenous enzymes such as polyphenol oxidase and peroxidase (Koley et al., 2009). mentioned that high h* value and low C* were responsible for the greenness of the pointed gourd; hence, packaging especially with polyethylene in combination with KMS or NaOCl would be able to keep the pointed gourd green during storage. Chemically treated fresh-cut papaya with MAP packaging also maintained the best values of L*, a*, and b* up to 25 days at storage, which is concurrent with the present study findings (Waghmare and Annapure, 2013).

### Nutritional attributes

#### Ascorbic acid

Ascorbic acid (30.8 mg/100 g) was depleted with the increase in shelf-life and storage temperature. Considering the highest shelf-life (15–23 days) at LT, non-perforated polyethylene and polypropylene (NPE and NPP) with KMS (C_3_) or without chemical (C_1_) secured a considerable amount of ascorbic acid (1.76, 1.75, 1.78, and 2.2 mg/100 g, respectively). However, NPE with KMS (2.06 mg/100 g) and no chemical (2.64 mg/100 g) also protected a substantial amount of ascorbic acid for up to 7–10 days while stored at RT (**Table 4**). Low O_2_ in refrigerated storage may protect the breakdown of ascorbic acid which can protect the oxidation and isomerization of the polyene chain associated with the degradation of β-carotene (Rodriguez-Amaya, 1997). Sahoo et al. (Sahoo et al., 2015) also mentioned such a lower depletion rate of ascorbic acid in refrigerated storage, but it was from perforated polypropylene with MAP, which only prolonged the shelf-life up to 16 days.

**Table 4:** Nutritional qualities of pointed gourd up to acceptance level under different postharvest treatment and packaging conditions.

Treatment	Ascorbic acid (mg/100 g)		β-Carotene (mg/100 g)	
	LT (4°C)	RT (30°C)	LT (4°C)	RT (30°C)
P_1_C_1_	2.83 ± 0.03b	3.76 ± 0.05a	7.2 ± 1.1d–f	34.7 ± 1.8a
P_1_C_2_	1.34 ± 0.02g	1.54 ± 0.22i	1.5 ± 0.4f	11.9 ± 0.6hi
P_1_C_3_	3.55 ± 0.05a	1.17 ± 0.12m	7.5 ± 1.8d–f	18.3 ± 1.1e–g
P_1_C_4_	1.11 ± 0.01h	1.49 ± 0.15j	13.5 ± 5.9b–d	8.7 ± 1.1j
P_2_C_1_	0.00 ± 0.00j	2.20 ± 0.00e	14.3 ± 0.6b–d	16.5 ± 0.8g
P_2_C_2_	0.00 ± 0.00j	1.76 ± 0.00h	10.9 ± 0.7c–e	16.5 ± 1.2g
P_2_C_3_	0.00 ± 0.00j	0.00 ± 0.00o	15.7 ± 3.1b–d	22.6 ± 0.9cd
P_2_C_4_	1.32 ± 0.00g	2.64 ± 0.00d	17.0 ± 6.4bc	12.7 ± 0.5h
P_3_C_1_	1.75 ± 0.05e	2.64 ± 0.00d	15.3 ± 0.5b–d	19.6 ± 1.2e
P_3_C_2_	0.00 ± 0.00j	1.77 ± 0.03h	2.8 ± 4.5ef	24.7 ± 1.2b
P_3_C_3_	1.76 ± 0.00e	2.06 ± 0.12f	10.8 ± 0.8c–e	6.3 ± 0.6k
P_3_C_4_	1.32 ± 0.00g	2.73 ± 0.11c	12.3 ± 4.7b–d	5.5 ± 0.4k
P_4_C_1_	0.00 ± 0.00j	1.21 ± 0.11l	10.6 ± 0.8c–e	25.3 ± 0.6b
P_4_C_2_	0.00 ± 0.00j	0.00 ± 0.00o	1.8 ± 0.3f	13.6 ± 0.9h
P_4_C_3_	0.00 ± 0.00j	0.00 ± 0.00o	11.7 ± 0.3cd	18.7 ± 0.6ef
P_4_C_4_	1.43 ± 0.05f	2.20 ± 0.00e	17.2 ± 5.4bc	12.5 ± 1.4h
P_5_C_1_	2.20 ± 0.10d	0.00 ± 0.00o	13.0 ± 0.3b–d	16.7 ± 1.3fg
P_5_C_2_	0.00 ± 0.00j	1.31 ± 0.01k	3.0 ± 4.5ef	18.3 ± 0.6e–g
P_5_C_3_	1.78 ± 0.02e	0.88 ± 0.00n	20.7 ± 0.9ab	25.6 ± 1.5b
P_5_C_4_	1.76 ± 0.00e	1.86 ± 0.11g	15.3 ± 11.4b–d	24.2 ± 1.5bc
P_6_C_1_	2.20 ± 0.00d	3.61 ± 0.09b	18.5 ± 0.7bc	16.9 ± 1.9fg
P_6_C_2_	2.37 ± 0.06c	0.00 ± 0.00o	10.7 ± 0.8c–e	8.4 ± 0.5j
P_6_C_3_	2.15 ± 0.05d	0.00 ± 0.00o	15.1 ± 7.6b–d	10.1 ± 0.4ij
P_6_C_4_	0.50 ± 0.05i	1.49 ± 0.15j	26.3 ± 11.1a	21.5 ± 1.2d
LS	**	*	**	**

P_1_ and C_1_, control; P_2_ and P_3_, perforated and non-perforated polyethylene (PPE and NPE, respectively); P_4_ and P_5_, perforated and non-perforated polypropylene (PPP and NPP, respectively); P_6_, brown paper (BP); C_2_, NaOCl; C_3_, KMS; C_4_, blanching.Numeric values (±) are standard deviation, and letters in the same row (a–l) are significant differences.*p < 0.05, **p < 0.01.

#### β-carotene

The accumulation of β-carotene (2.2 mg/100 g) increased with prolonged storage period and temperature. At LT storage, non-perforated polyethylene and polypropylene were confined to the formation of β-carotene (2.8 and 3.0 mg/100 g, respectively) with NaOCl (C_2_) for up to 17 days. Meanwhile, NPE with KMS was able to retard β-carotene formation at an intermediate level (10.8 mg/100 g) but prolonged the storage period up to 23 days. On the other hand, NPE and KMS inhibited storing β-carotene remarkably (6.3 mg/100 g) up to 10 days at RT storage conditions (**Table 4**). NPE with KMS or NaOCl might be able to retain ascorbic acid and retard the accumulation of β-carotene in pointed gourd during extended storage durations. Lower respiration and ethylene production, subsequently reducing ethylene action and delaying ripening and senescence due to the modified gas atmosphere, could be the possible reasons (Haile, 2018).

### Multivariate analysis

#### Correlation matrix

**Figure 1:**
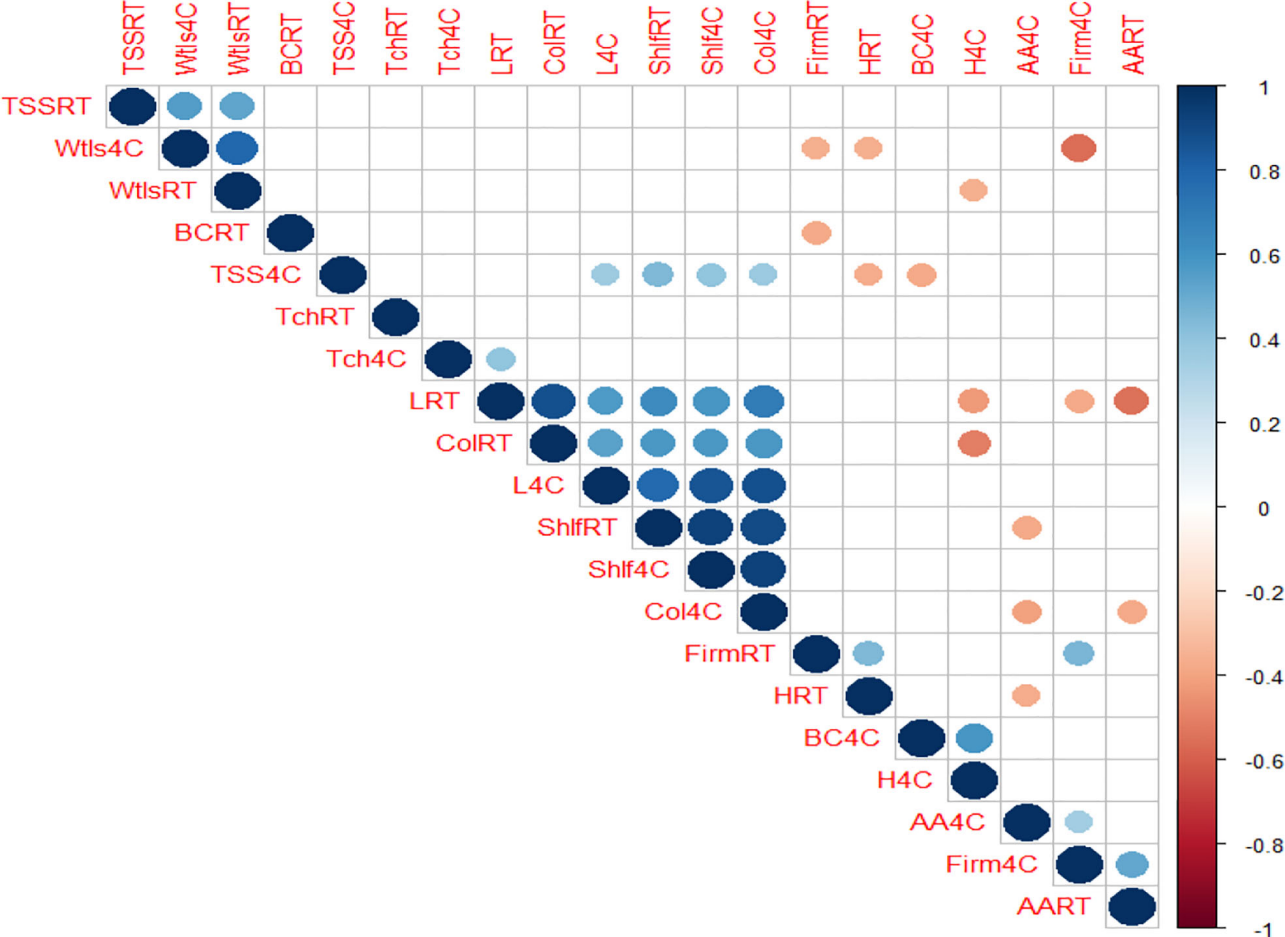
Correlation matrix in between and among physiochemical, colorimetric, and nutritional parameters studied for pointed gourd quality storage. Shlf, shelf-life; Firm, firmness; Wtlos, weight loss; TSS, total soluble solid; Tch, total chlorophyll; L, lightness; H, hue; Col, chroma; AA, ascorbic acid; BC, β-carotene; 4C, refrigerated at 4°C at 45% RH; RT, room temperature 30 ± 2°C at 65–75% RH.

Pearson’s correlation was assessed to prepare a correlation matrix and to detect the interrelationship among the studied physiochemical and nutrition attributes of pointed gourd during LT and RT storage. Under LT condition, positive correlations across TSS, L*, C*, shelf-life, β-carotene (BC), h*, and ascorbic acid (AA) and firmness were observed, while firmness (weight loss was strong), BC, TSS, AA, and C* were negatively correlated. This relationship indicated that TSS, L*, and C* increased with shelf-life and that β-carotene conversion increased the h* value. Firmness declined with the breakdown of AA and rapid moisture loss during LT storage. Meanwhile, at RT storage conditions, L*, C*, and shelf-life were also positively correlated. Firmness was positively correlated with h*, while it was negatively correlated with BC. However, L* and AA had a strongly negative relationship under RT storage conditions (**Figure 1**). This suggested that the trend of declined firmness was addressed with the decreased h* value and increased β-carotene conversion. The breakdown of ascorbic acid leads to an increase in lightness under RT storage conditions. Therefore, the correlation matrix indicated that the plausible retention of ascorbic acid, TSS, and firmness would maintain the green color along with a longer shelf-life of the pointed gourd fruit.

### Principal component analysis

**Figure 2:**
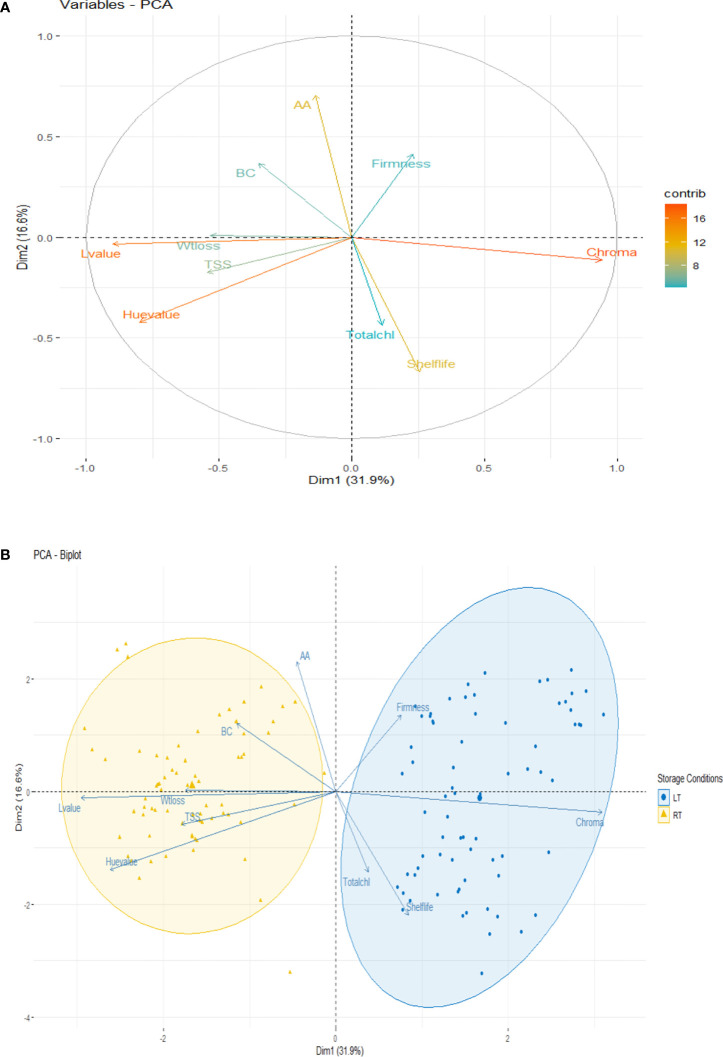
Principal component analysis (PCA) of the physiochemical, colorimetric, and nutritional parameters studied for pointed gourd quality storage. **(A)** PCA of the variables showing their major contribution. **(B)** PCA–biplot analysis representing the performance of quality parameters at low and room temperature. Wtlos, weight loss; TSS, total soluble solid; Totalchl, total chlorophyll; Lvalue, lightness (L*); Hvalue, Hue (h*); chroma, C*; AA, ascorbic acid; BC, β-carotene; LT, refrigerated at 4°C at 45% RH; RT, room temperature 30 ± 2°C at 65–75% RH.

The above-mentioned result showed that the average physiochemical and nutritional attributes and color coordinates of the refrigerated (LT) and ambient (RT) storage of pointed gourd fruits in different packaging and postharvest treatments were correlated with each other (**Figure 1**). In addition, the results shown in **Tables 1**–**4** indicated that the color and nutrient composition of stored pointed gourd were significantly affected by packaging and postharvest treatment. However, a mere visual inspection cannot properly detect such differences between packaging materials as well as postharvest treatments. Therefore, all the studied physiochemical and nutrient compositions (dependent variables) were analyzed using PCA to explore the relative variability within the different stored pointed gourd samples for effective packaging and postharvest treatment selection. As observed, the two principal components (dimension 1 and dimension 2) explained 48.5% of the total variations. L*, h*, and C* were noticed as strong, shelf-life and ascorbic acid were noticed as intermediate, and weight loss, firmness, TSS, total chlorophyll, and β-carotene had low contributions on post-harvest acceptance in pointed gourd storage (**Figure 2A**). Dim1 explained 31.9% and Dim2 explained 16.6% of the total variability among the variables generated from LT and RT storage conditions. Dim1 could be positively associated in a chronological way as follows: chroma< shelf-life< firmness< total chlorophyll, while negatively associated with hue< L< AA< TSS< BC< weight loss for LT storage. On the other side, Dim2 could be positively associated chronologically with AA< firmness< BC, whereas it could be negatively associated with hue< chroma< L< shelf-life< total chlorophyll< TSS< weight loss for RT storage (**Figure 2B**). The results of the PCA analysis revealed that LT and RT storage conditions had different effects on pointed gourd storability.

## Conclusion

Based on the results, it has been revealed that at both LT and RT storage conditions, NPE along with KMS possibly reduced the O_2_ and increased CO_2_ and restricted the enzymatic activity of respiration and transpiration. Thus, the storage life of the pointed gourd was prolonged up to 23 and 10 days under LT and RT conditions, respectively, with lower physiochemical, colorimetric, and nutritional loss. Although greenness indicating higher h* and lower C* values was better in PPE and PPP with NaOCl, it was able to provide only 6 days instead of 10 days during RT storage. The PCA study suggested five contributing variables (L*, C*, h*, shelf-life, and ascorbic acid) recognized as the most important postharvest quality attributes, and those were subject to be maintained in non-perforated polyethylene packing with KMS (0.05%) both at LT and RT storage conditions. Therefore, NPE in combination with KMS could be adapted for postharvest quality improvement of the pointed gourd for 23 days at LT and 10 days at RT storage conditions.

## Data availability statement

The original contributions presented in the study are included in the article/supplementary material. Further inquiries can be directed to the corresponding authors.

## Author contributions

JH conceived the idea of the study, designed the experiment, analyzed the data, conducted the work, and wrote the manuscript. FJ helped to collect data and in its interpretation. MR contributed to the research work and prepared the manuscript. US assisted in data analysis, reviewed the manuscript, and made suggestions for improvement. IM and YO edited the manuscript, provided valuable suggestions during the experiment, and also provided valuable support and guidance in preparing the manuscript. US, SE, KSG, and RM edited the manuscript, provided valuable suggestions during the experiment, and also provided valuable support and guidance in preparing the manuscript. All authors contributed to the article and approved the submitted version.

## Conflict of interest

The authors declare that the research was conducted in the absence of any commercial or financial relationships that could be construed as a potential conflict of interest.

## Publisher’s note

All claims expressed in this article are solely those of the authors and do not necessarily represent those of their affiliated organizations, or those of the publisher, the editors and the reviewers. Any product that may be evaluated in this article, or claim that may be made by its manufacturer, is not guaranteed or endorsed by the publisher.
